# Patient Initiated Follow-Up (PIFU) Within Adult Secondary Care Mental Health Services

**DOI:** 10.1192/bjo.2022.368

**Published:** 2022-06-20

**Authors:** Andrew Wilkinson, Pavan Srireddy

**Affiliations:** NHS Greater Glasgow and Clyde, Glasgow, United Kingdom

## Abstract

**Aims:**

The traditional ‘one size fits all’ model within secondary care mental health (MH) settings of regular appointments scheduled by a clinician at defined intervals isn't always responsive to an individual's changing needs. Previous reviews have shown significant levels of patient and clinician satisfaction with Patient initiated models of review in a variety of healthcare settings but its use within secondary care MH settings has been relatively limited. We describe the development and implementation of a Patient initiated follow-up (PIFU) pathway within MH services in NHS Greater Glasgow and Clyde (GG&C).

**Methods:**

The pathway was developed by a small working group of clinicians with input from local management and eHealth colleagues with an emphasis on the principles of Realistic Medicine. There was input from peer support workers and the Mental Health network, a local service user organisation, into the development of the pathway. The pathway underwent a ‘test of implementation’ within three adult CMHT's with support from the development group. Feedback from the test sites was used to modify the pathway and ultimately support the wider rollout of the model across all seventeen CMHTs within NHS GG&C over the course of 2021. Formal evaluation of the pathway, including patient and clinician satisfaction, service utilisation as well as safety measures, is due to be undertaken at 12 months after full implementation.

**Results:**

The tests of implementation identified a range of factors that needed to be considered as part of the introduction of a PIFU model into MH settings.

Patient choice and shared decision making along with other clinical factors such as level of insight, availability of other supports, shared risk assessments and current clinical need were identified as relevant patient related factors. Clinician related factors included concerns about applicability within MH settings, perception of risk, increase in workload and appropriate identification of suitable patients. Regular meetings between the clinicians in the test sites and members of the development group as part of the implementation process helped address clinicians concerns and ultimately supported uptake of the model.

**Conclusion:**

Our experience highlighted the potential for a personalised approach to care planning in empowering patients have a more active role in the way they access services as part of their recovery journey. It also highlighted patient and clinician related factors that need to be considered for a successful adoption of the model.

